# Virtual simulation in nursing education in Latin America and the Caribbean: A bibliometric study*

**DOI:** 10.1590/1518-8345.7261.4422

**Published:** 2025-03-14

**Authors:** Agostinho A.C. Araújo, Lucas Gardim, Sara Soares dos Santos, Ítalo Rodolfo Silva, Manoel Carlos Neri da Silva, Simone de Godoy, Isabel Amélia Costa Mendes

**Affiliations:** 1 Universidade de São Paulo, Escola de Enfermagem de Ribeirão Preto, PAHO/WHO Collaborating Centre for Nursing Research Development, Ribeirão Preto, SP, Brazil; 2 Scholarship holder at the Conselho Nacional de Desenvolvimento Científico e Tecnológico (CNPq), Brazil; 3 University of Alberta, Faculty of Nursing, Edmonton, AB, Canada; 4 Universidade Federal do Rio de Janeiro, Escola de Enfermagem Anna Nery, Rio de Janeiro, RJ, Brazil; 5 Conselho Federal de Enfermagem, COFEn, Brasília, DF, Brazil

**Keywords:** Nursing Education, Nursing, Teaching, Nursing Students, Virtual Reality, Simulation Training

## Abstract

to examine the characteristics of scientific production in Latin America and the Caribbean regarding virtual simulation in nursing education.

a bibliometric study conducted in five stages: research design, compiling the bibliometric data, analysis, visualization, and interpretation; and based on the recommendations of the Preferred Reporting Items for Bibliometric Analysis (PRIBA). The search was conducted in the Latin American and Caribbean Health Sciences Literature (LILACS). VOSviewer version 1.6.20 was used for bibliometric analysis. Bradford’s and Zipf’s Laws were applied to interpret the data, considering the temporal dynamics of publications, an analytical approach to the structure of the selected studies and their repercussions in virtual simulation in nursing education in Latin America and the Caribbean.

579 studies were identified, of which 46 were included in the final sample. The studies included were published between 1994 and 2022, and only Brazil, Chile, Colombia, and Cuba conducted research related to virtual simulation. The semantic map resulted in five clusters, highlighting “simulation training” as the most important term.

our findings suggest a projected increase in Scholarly publications on virtual simulation in nursing education in Latin America and the Caribbean in the coming years.

## Highlights

**(1)** Virtual simulation connects theory and practice in nursing education.**(2)** Only Brazil, Chile, Colombia, and Cuba conducted research on the subject. **(3)** Brazilian journals lead scientific production on virtual simulation. **(4)** Scientific production on the subject has increased.

## Introduction

From their undergraduate education, students need to develop the competencies required to deliver quality and safe healthcare^(1-2)^. With the rapid advancements in health technologies, there is an increased expectation for investment in nursing education, particularly in the pedagogy that supports practice^(3-4)^. As a result, virtual simulation has become essential in addressing educational plans.

Virtual simulation is an educational strategy that allows the approximate reproduction of real situations in a controlled environment, enhancing the development of essential skills in clinical practice^(5-6)^. From a historical perspective, with the first teaching methods adopted in nursing education, students developed their clinical skills by performing procedures directly on their peers or patients. However, this practice poses risks for both students and patients^(7)^. As nursing advances as a profession and a science, intrinsic technological development enhances the progress of education, and, as a result, rudimentary teaching practices are no longer adopted in different contexts^(7-8)^.

Among different modalities of virtual simulation, clinical simulation emerge as a successful teaching and learning strategy that gives students the opportunity to practice their clinical skills without harming or risking patients, enhancing the qualification of nursing education^(9-10)^. When applied to nursing education, clinical simulation fosters self-confidence and satisfaction in nursing students from their earliest days of education, during their undergraduate studies and throughout the evolution of their education^(8,11-12)^, which reveals the indispensability of clinical simulation environments in the educational process^(13)^.

Although scientific evidence demonstrates that clinical simulation in nursing education to achieve positive outcomes related to clinical practice (especially as an educational strategy that provides a transition from theory to practice)^(14-15)^, many Higher Education Institutions (HEIs) still do not use clinical simulation in their educational process^(16)^. This reality becomes even more alarming when considering the incorporation of virtual simulation in Latin America and the Caribbean. While it enhances the quality of clinical education, its high cost presents significant challenges for both implementation and long-term sustainability in nursing education.

According to the Economic Commission for Latin America and the Caribbean (ECLAC), 2023 has been predicted as a challenging year for the countries in the region, given the low economic growth caused by high inequality and poor administration^(17)^. In these countries, low economic growth directly reduces the investment in education and, consequently, access to technologies. Therefore, the incorporation of virtual reality in nursing education becomes a major challenge for the region.

Virtual simulation is an important strategy in nursing education to improve nursing students’ knowledge, skills, and attitudes. Therefore, considering the importance of understanding the panorama and trends of virtual simulation for advancing nursing education in the digital era, this study aimed to examine the characteristics of scientific production in Latin America and the Caribbean regarding virtual simulation in nursing education.

## Method

### Study type

This bibliometric study was conducted in five stages, namely: research design, compiling the bibliometric data, analysis, visualization, and interpretation^(18)^; and was reported following the recommendations of the Preferred Reporting Items for Bibliometric Analysis (PRIBA)^(19)^.

We used the bibliographic coupling and co-word analysis methods^(18)^. Thus, considering the scenario in Latin America and the Caribbean, the following research questions were outlined:

How has scientific production on virtual simulation in nursing education evolved?

Which scientific journals have published research on virtual simulation in nursing education?

What are the modalities of virtual simulation in nursing education?

How did the keywords co-occur in studies related to virtual simulation in nursing education?

### Data sources

Our study was conducted in the Latin American and Caribbean Literature in Health Sciences (LILACS) database as it is the most important and comprehensive specialized database related to healthcare in the region, with scientific and technical literature from 26 Latin American and Caribbean countries with free and open access. This database contains around 1 million records in 910 journals over a 38-year history^(20)^. In comparison with other databases, such as the Medical Literature Analysis and Retrieval System Online via the National Library of Medicine (MEDLINE), Cumulative Index to Nursing and Allied Health (CINAHL), Web of Science, Embase, and Scopus, although not mutually exclusive, only LILACS is sensitive to the context of the study, given its specificity in encompassing the most extensive scientific collection limited to the region.

### Eligibility criteria

Studies that met the following criteria were included: 1) primary studies on virtual simulation in nursing education; 2) studies from Latin American and Caribbean countries; and 3) studies published in Portuguese, Spanish, or English (languages the authors speak). Studies conducted from the perspective of nursing educators regarding virtual simulation were excluded. This bibliometric study did not adopt a timeframe. However, a selection of studies published up to the year 2022 was established, considering this to be the last full year during the development of the study, which makes bibliometric analysis with a focus on dynamics feasible; in addition, gray literature was not considered.

### Search strategy

A pilot search was carried out in April 2023 in order to identify the terms registered in the Health Sciences Descriptors (DeCS) that were most suitable for developing the search strategy. The search was conducted on May 5th of the same year, applying the following search strategy: ((mh:(“Realidade Virtual”)) OR (mh:(“*RealidadVirtual*”)) OR (mh:(“*Virtual Reality*”)) OR (“Realidade Virtual”) OR (“*RealidadVirtual*”) OR (“*Virtual Reality”*) OR (mh:(”Treinamento por Simulação”)) OR (mh:(“*Computer Simulation*”)) OR (mh:(“*Entrenamiento Simulado*”)) OR (“Treinamento por Simulação”) OR (“*Computer Simulation*”) OR (“*Entrenamiento Simulado*”) OR (mh:(“Simulação por Computador”)) OR (mh:(“*Computer Simulation*”)) OR (mh:(“*Simulación por Computador*”)) OR (“Simulação por Computador”) OR (“*Computer Simulation*”) OR (“*Simulación por Computador*”) OR (“Simulação Virtual”) OR (“*Simulación Virtual*”) OR (“*Virtual Simulation*”)) AND ((mh:(“Educação em Enfermagem”)) OR (mh:(“*Educación en Enfermería*”)) OR (mh:(“*Education, Nursing*”)) OR (mh:(“*Nursing Education*”)) OR (“Educação em Enfermagem”) OR (“*Educación en Enfermería*”) OR (“*Education, Nursing*”) OR (“*Nursing Education*”) OR (mh:(“Educação”)) OR (mh:(“*Educación*”)) OR (mh:(“*Education*”)) OR (Educa*) OR (“*Educación*”) OR (“*Education*”) OR (mh:(“Enfermagem”)) OR (mh:(“*Enfermería*”)) OR (mh:(“*Nursing*”)) OR (“Enfermagem”) OR (“*Enfermería*”) OR (“*Nursing*”) OR (Enfermeir*) OR (Nurse*) OR (Enfermer*)). The search strategy was developed with the support of an experienced librarian.

In order to reduce the inclusion of studies that diverged from the proposed method (especially concerning outliers - studies that differ significantly from the others in that their presence can reduce the validity of the results), the studies found were exported to the Rayyan platform for independent screening by two reviewers^(21)^, following the methodological framework^(18)^. The screening, eligibility, and inclusion of the studies were based on reading the titles and abstracts, with the objective and research questions as guiding principles. Any discrepancies during the analysis were resolved by a third reviewer, an expert in the field, who assisted in making the final selection of studies.

### Bibliometric indicators

The final sample was exported to Microsoft Excel^®^ for descriptive analysis of the selected studies. Bibliometric analysis was then carried out using VOSviewer, version 1.6.20. This software was chosen since it allows the construction of bibliometric networks that can include journals, groups of researchers, citation networks, authorship networks, co-authorship, and groups of the most prevalent words^(22)^. The keywords and their variations were presented in only one format, i.e., the root word was replaced by its most complete version, considering its entry in the DeCS. This strategy was applied to avoid duplicate data in the final sample, according to the methodological framework^(18)^.

Two bibliometric laws were used to analyze the data: Bradford’s and Zipf’s. Bradford’s Law examines the productivity of journals, classifying their relevance based on the number of articles published on a specific topic. On the other hand, Zipf’s Law measures the frequency of words in texts, enabling them to be ordered and analyzed according to the research focus^(23)^. In addition, the annual evolution of publications and the type of simulation used in articles published in Latin America and the Caribbean on virtual simulation in nursing education were considered.

Over time, a descriptive analysis of scientific production on virtual simulation in nursing education was presented. The number of publications was detailed according to year of publication, scientific journals, and simulation modalities. In addition, for the bibliometric analysis of the co-occurrence of keywords, the data was presented in semantic maps in order to detail the cognitive structure of the field proposed for the scope of this study.

The purpose of the bibliometric study is to describe a field^(18)^, defined in this study as virtual simulation in nursing education in Latin America and the Caribbean. Thus, the analysis focused on dynamics in order to interpret the years in which the studies were published, based on a timeline that considered events that justify the increase or decrease in the production of virtual simulation in nursing education. In addition, the journals with the highest number of publications on the subject were analyzed, which may be relevant for researchers interested in virtual simulation issues in nursing education.

An analytical approach was used to interpret the structure of the selected studies, enabling a clear understanding of the relationships between key structural elements (such as scientific journals and simulation modalities) and their influence on the field within the proposed scope. Next, the semantic map was defined according to the clusters of the co-occurrence collaboration of the keywords. For the keyword co-occurrence analysis, VOSviewer was configured to consider at least two occurrences of the same keyword.

## Results

**Figure 1 -:**
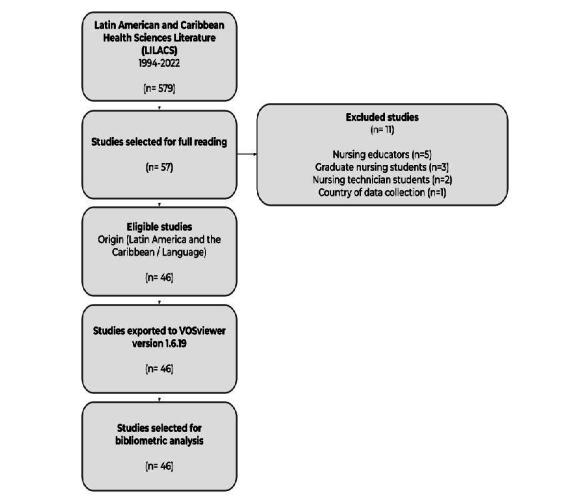
Preferred Reporting Items for Bibliometric Analysis (PRIBA)

A total of 579 studies were identified, of which 57 were selected for full reading. After applying the eligibility criteria, 46 studies composed the final sample^(24-69)^, as shown in Figure 1.

**Figure 2 -:**
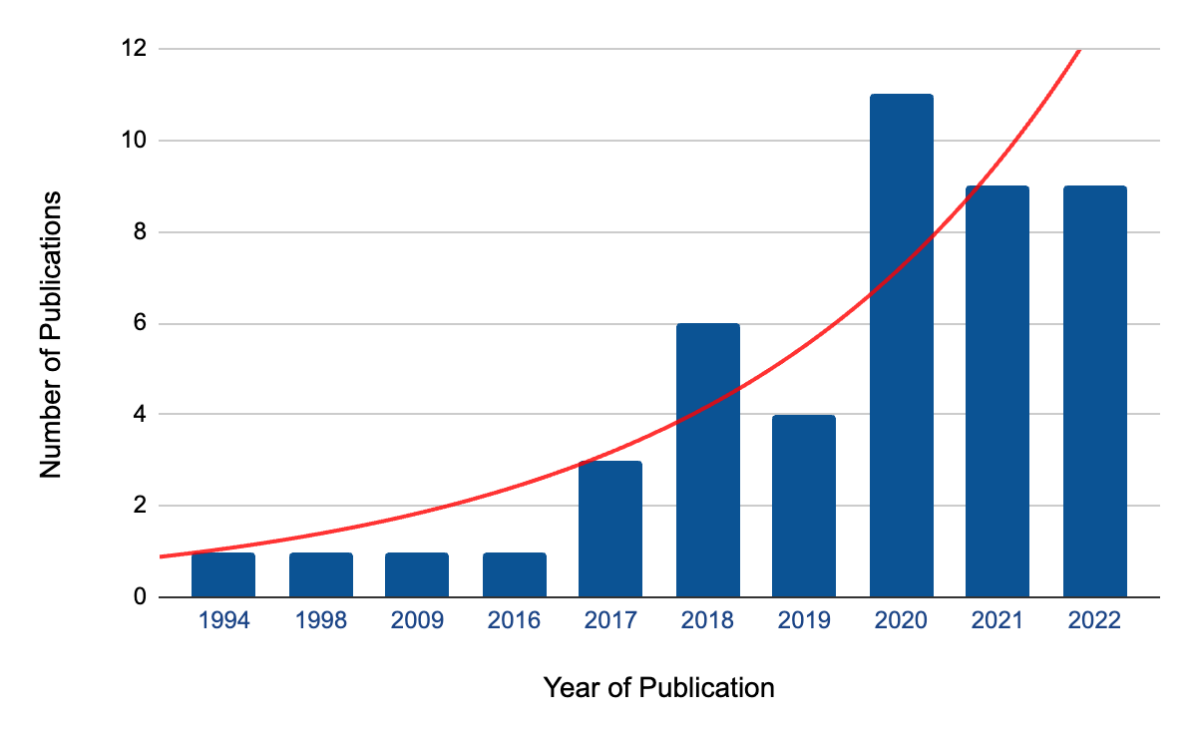
Number of articles published annually in Latin America and the Caribbean on virtual simulation in nursing education. Ribeirão Preto, SP, Brazil, 2023

This bibliometric study analyzed 46 scientific articles on virtual simulation in nursing education in Latin America and the Caribbean. The sample has an average of six authors per publication, and there has been a notable increase in scientific production in this area, from an average of 0.17 articles per year between 1994 and 2016 to 4.33 between 2017 and 2019 and 9.6 between 2020 and 2022, as shown in Figure 2.

Only four countries in Latin America and the Caribbean have conducted research on virtual simulation in nursing education. Brazil led with approximately 84.78% (n=39) of the total studies^(24-39,41-51,53-62,64-65)^. Chile followed with about 6.52% (n=3)^(63,66-67)^, while Colombia and Cuba each contributed 4.35% (n=2)^(40,52,68-69)^.

**Figure 3 -:**
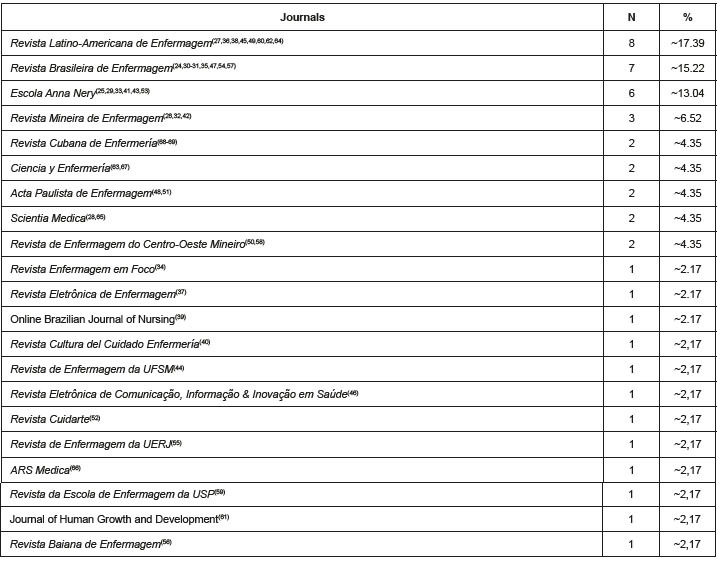
Journals in which articles from Latin America and the Caribbean on virtual simulation in nursing education were published. Ribeirão Preto, SP, Brazil, 2023

Figure 3 shows the journals that published the most studies related to the subject, predominantly from Brazil, with an emphasis on *Revista Latino-Americana de Enfermagem*, with 17.39% (n=8)^(27,36,38,45,49,60,62,64)^, *Revista Brasileira de Enfermagem*, with 15.29% (n=7)^(24,30-31,35,47,54,57)^, and *Escola Anna Nery*, with 13.04% (n=6)^(25,29,33,41,43,53)^.

**Figure 4 -:**
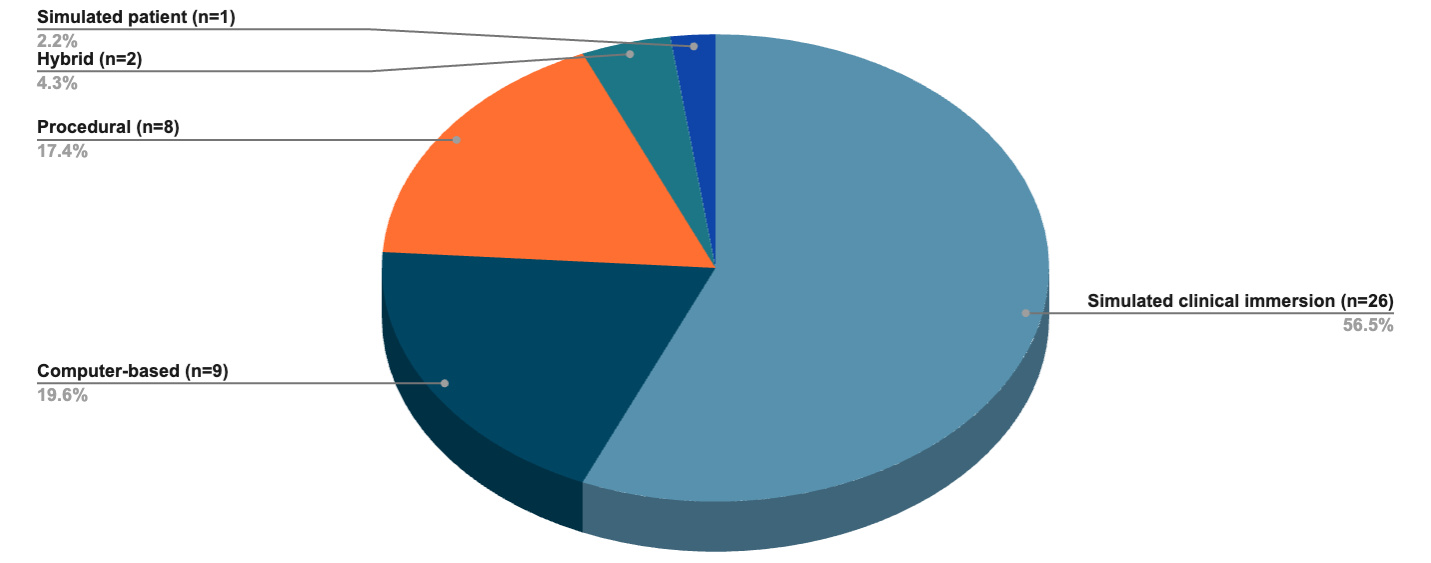
Simulation modality used in articles published in Latin America and the Caribbean on virtual simulation in nursing education. Ribeirão Preto, SP, Brazil, 2023

Based on a conceptual framework^(70)^, the scenarios identified in the sample were classified according to simulation modalities (Figure 4).

**Figure 5 -:**
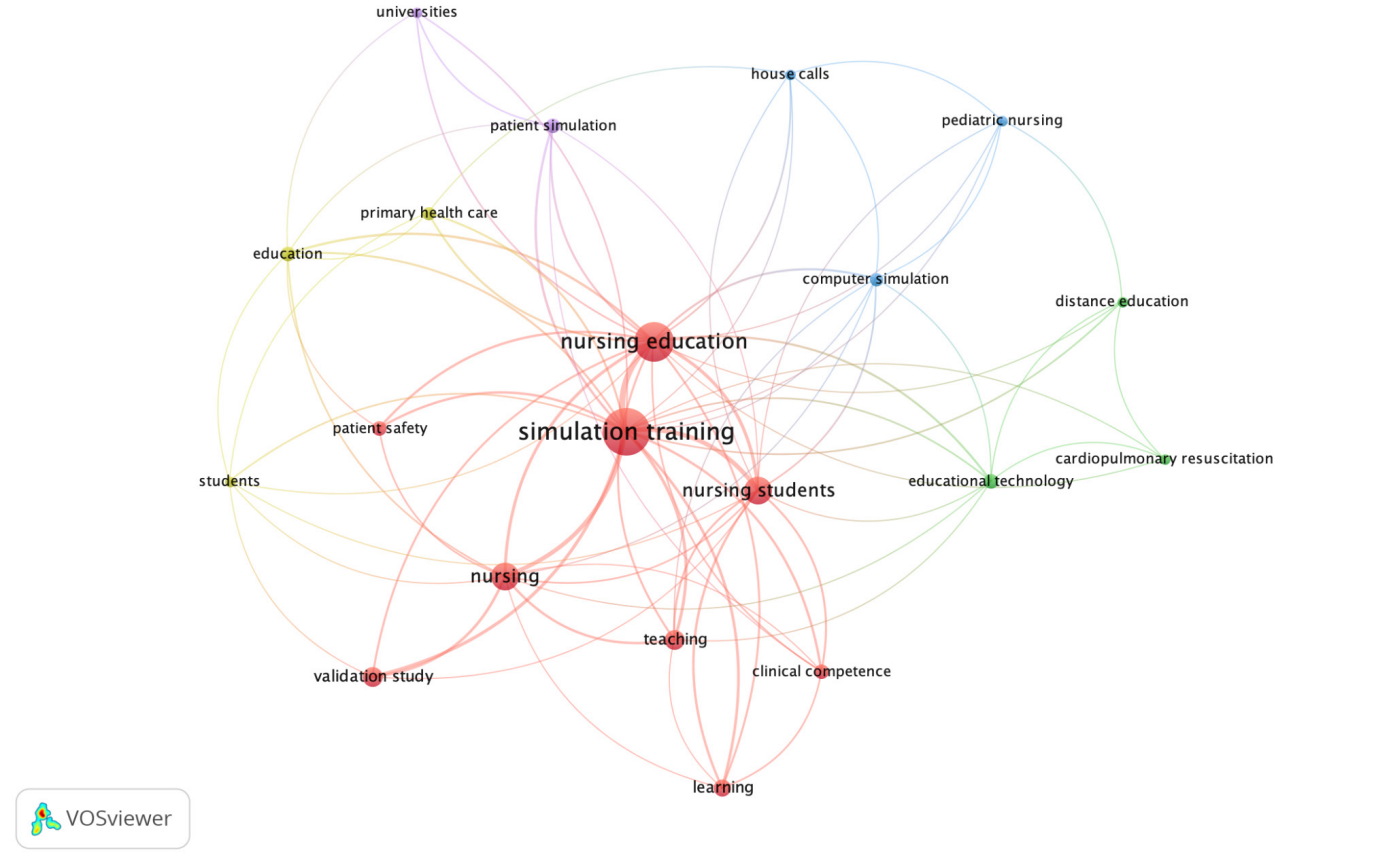
Analysis of co-occurrence of keywords by authors of studies published in Latin America and the Caribbean on virtual simulation in nursing education. Ribeirão Preto, SP, Brazil, 2023

The authors defined the cognitive structure using keywords that summarized the focus of their studies. Thus, 272 keywords were found, grouped according to co-occurrence, with at least two mentions. As illustrated in Figure 5, the bibliometric network represents the co-occurrence of keywords, showing the number of terms in common using circles and lines. The size of the circles and the thickness of the lines are proportional to the number of terms cited so that the connections between the terms indicate their relationships.

The term “simulation training” (n=38) had the highest frequency and link strength, connected to other terms such as “nursing education” (n=27), “nursing students” (n=14), and “nursing” (n=14). Other highlighted areas include: “clinical competence” (n=4), “patient simulation” (n=4), “education” (n=4), and “patient safety” (n=4). Figure 5 underscores the significance of practical and simulated training in developing essential clinical skills in nursing, highlighting its intersection with innovative teaching methods and patient safety.

## Discussion

In the face of a significant context, Latin America and the Caribbean, the progressive increase in publications on virtual simulation in nursing education may be a consequence of global phenomena, either of a procedural or emergency nature. The first relates to the collective maturing of a profession or area of knowledge toward objects of interest that are dear to them. The second relates to emerging and detailed issues that can affect the apparent natural order of social processes, which includes the pattern of scientific publication.

At the operational level, scientific results reveal the need for greater and better connections between practical reality and the theoretical approach in the teaching-learning process. Thus, scientific production is expected to advance towards the development of better evidence on mechanisms and strategies to facilitate training that is coherent with the social demands already perceived and problematized in the course of the student’s training, which does not exclude the reality of health and, consequently, of nursing^(71-72)^.

In nursing education, when associated with active methodologies, virtual simulation is a valuable strategy for connecting the real and virtual worlds during the student’s teaching-learning process, enhancing student performance and the achievement of objectives by professors and HEIs. Virtual simulation allows students to be transported to totally parallel and virtual realities generated by computer intelligence, where they can safely explore and live experiences outside of the objective reality. These experiences stimulate senses such as sight, hearing, and sometimes even touch and smell. It is from this reality, therefore, that nursing and other areas of knowledge have been interested in researching clinical simulation^(4)^.

Teaching simulations can vary in context (basic life support, nursing care for women, and home visits) and level of immersion. This study analyzed virtual simulations such as telesimulation, serious games, and immersive virtual reality. Another modality was simulated clinical immersion, a model adopted more frequently and which recreates clinical scenarios with a high level of realism, involving patient care, complex equipment, actors, and a significant flow of data^(70)^.

Another form of simulation is procedural, which allows specific technical skills to be practiced in certain procedures, such as peripheral venous catheterization and the safe administration of drugs and vaccines. Finally, there is the simulated patient, in which an actor plays the role of an actual patient to train and manage clinical and emotional needs and conditions. These strategies are effective for developing attitudinal aspects in the quality of cognitive, behavioral, and psychomotor skills, as well as affective aspects of students in a simulated environment^(73)^. In this sense, with the need to develop a culture of innovation throughout the academic infrastructure, the teaching environment has the potential to inspire the development of leadership, a key competence for nursing^(74)^.

Regarding the emerging and detailed issues that affect social dynamics, the data from this research was sensitive to the significant increase in studies published during the COVID-19 pandemic. This situation has highlighted the need for better technological adaptations to maintain the training process. As a consequence, better responses based on science and speed in scientific production are needed. This reiterates the dynamics of scientific publishing as a constantly evolving process shaped by the interaction between technological advances, the demands of society, and the practices established in the community^(75)^.

As a leader in the production of virtual simulation in Latin America and the Caribbean, Brazil saw a 32.2% increase in the number of scientific articles in 2020 compared to 2015, according to data consolidated by the Observatory of Science, Technology, and Innovation (OCTI) and the Center for Management and Strategic Studies (CGEE)^(76)^. As highlighted in the results, this study showed that Brazilian productions are the majority, amounting to more than 80%. This may be due to the demands of a continental country and its graduate training structure, since it has 39 doctoral courses in nursing, whereas Chile (the country in second place in terms of production on virtual simulation) has two doctoral courses. This scenario is attested to by the understanding that scientific production is mainly developed in the context of universities and their graduate courses^(77)^.

The apparent plurality of scientific journals that have published on virtual simulation (identified in our study in a total of 21 journals) is not, however, in line with the reality in Latin America and the Caribbean, as it does not represent or even come close to the totality of journals in this region. As a numerical illustration, the Ibero-American Network of Scientific Editing in Nursing (RedEDIT) has 72 journals published in Latin America. This number of journals does not represent all the journals in this region, since not all of them are associated with the aforementioned network^(78)^. Furthermore, the concentration of publications in only three journals demonstrates the indispensability of investments in scientific research as an incentive to develop evidence related to virtual simulation in nursing.

The bibliometric network is an important imaging mechanism for the reality under investigation. Thus, the terms “simulation training”, “nursing education” and “nursing students”, although prospected in the theme intended in this study, reveal the problematized nature of the object, as they demonstrate the importance of strategies that associate technologies and principles of active methodology towards the teaching-learning process capable of provoking new experiences for students, based on the reality of patient care which is permeated by uncertainties and risks. In peripheral fields of the bibliometric network, it is possible to see terms related to specific knowledge concerning advanced practices, for example. These include the terms “cardiopulmonary resuscitation”, “clinical competence”, “pediatric nursing”, and “patient safety”. Thus, the semantic map highlights the importance of simulation training and addressing related issues, pointing to other emerging concepts that can potentially shape nursing education in the future.

Although the leading Latin American and Caribbean database, LILACS, was used in this study, it is important to note that some scientific journals in the region are not indexed in this database. This limitation may have affected the identification of potential studies. Additionally, this study did not include research published in French. While French is relevant to the region, the authors were unable to access studies in this language due to their lack of proficiency. Studies in French could provide information on the scientific production of virtual simulation in nursing education within Latin America and the Caribbean.

Although the study is related to undergraduate nursing education and, therefore, towards the process of generalist training, virtual simulation can provide better possibilities for theoretical and technical-scientific in-depth study of knowledge specific to nurses’ professional practice. Thus, despite the disparities in scientific production, this practice should be recommended in the context of nursing education for the advancement of the profession.

## Conclusion

The panorama of scientific production on virtual simulation in nursing education in Latin American and Caribbean countries was shown, with a predominance of Brazilian scientific production, in which the *Revista Latino-Americana de Enfermagem* (RLAE) takes the lead. The results reinforce the understanding of “simulation training” as a valuable strategy for connecting theory and practice during the teaching-learning process, corroborating the development of competencies in nursing students. The study underscores the importance of virtual simulation research for nursing education, highlighting a trend towards increased development of studies related to virtual simulation with a focus on simulated clinical immersion. Furthermore, it emphasizes the significant opportunities for international collaboration and resource sharing between researchers, promoting a comprehensive and collaborative approach to advances in this field. However, economic factors are the main barrier to implementing and sustaining virtual simulation in nursing education, as well as developing new scientific evidence to address various scenarios and contexts.
