# Supplementary material for: Virtual simulation in nursing education in Latin America and the Caribbean: A bibliometric study
Source: Rev Lat Am Enfermagem. 2025 Mar 14;33:e4422. doi: 10.1590/1518-8345.7261.4422 (PMC11912820; doi:10.1590/1518-8345.7261.4422)
Supplement: Translation

# Translation: Simulação virtual na educação em enfermagem na América Latina e Caribe: estudo bibliométrico*

**DOI:** 10.1590/1518-8345.7261.4423

**Authors:** Agostinho A.C. Araújo, Lucas Gardim, Sara Soares dos Santos, Ítalo Rodolfo Silva, Manoel Carlos Neri da Silva, Simone de Godoy, Isabel Amélia Costa Mendes

**Affiliations:** 1 Universidade de São Paulo, Escola de Enfermagem de Ribeirão Preto, Centro Colaborador da OPAS/OMS para o Desenvolvimento da Pesquisa em Enfermagem, Ribeirão Preto, SP, Brasil; 2 Bolsista do Conselho Nacional de Desenvolvimento Científico e Tecnológico (CNPq), Brasil; 3 University of Alberta, Faculty of Nursing, Edmonton, AB, Canadá; 4 Universidade Federal do Rio de Janeiro, Escola de Enfermagem Anna Nery, Rio de Janeiro, RJ, Brasil; 5 Conselho Federal de Enfermagem, COFEn, Brasília, DF, Brasil

**Keywords:** Educação em Enfermagem, Enfermagem, Ensino, Estudantes de Enfermagem, Realidade Virtual, Treinamento por Simulação

## Abstract

examinar as características da produção científica da América Latina e Caribe a respeito da simulação virtual na educação em enfermagem.

estudo bibliométrico conduzido em cinco etapas: delineamento de pesquisa, compilação de dados bibliométricos, análise, visualização e interpretação; e reportado a partir das recomendações do *Preferred Reporting Items for Bibliometric Analysis* (PRIBA). A busca foi conduzida na Literatura Latino-Americana e do Caribe em Ciências da Saúde (LILACS). Utilizou-se o *VOSviewer version* 1.6.20 para análise bibliométrica. As Leis de Bradford e Zipf foram aplicadas para a interpretação dos dados, na medida em que foi considerada a dinâmica temporal de publicações, abordagem analítica da estrutura dos estudos selecionados e suas repercussões no campo da simulação virtual na educação em enfermagem na América Latina e Caribe.

foram localizados 579 estudos, dos quais 46 compuseram a amostra final. Os estudos incluídos foram publicados entre 1994 e 2022, sendo que apenas Brasil, Chile, Colômbia e Cuba realizaram pesquisas relacionadas à simulação virtual. O mapa semântico resultou em cinco *clusters*, destacando “treinamento por simulação” como o termo de maior força.

observa-se tendência crescente no desenvolvimento de estudos relacionados à simulação virtual na educação em enfermagem na América Latina e Caribe.

## Destaques

**(1)** A simulação virtual conecta teoria e prática na educação em enfermagem. **(2)** Apenas Brasil, Chile, Colômbia e Cuba realizaram pesquisas sobre o tema. **(3)** Os periódicos brasileiros lideram a produção científica sobre simulação virtual. **(5)** Observa-se crescimento da produção científica relacionada ao tema.

## Introdução

Ainda no contexto da graduação, faz-se indispensável que os estudantes desenvolvam competências que os habilitem para o empreendimento da assistência em saúde com qualidade e segurança^(1-2)^. No que diz respeito à enfermagem, tendo em vista o crescente progresso das tecnologias em saúde, espera-se maior investimento aplicado à pedagogia subjacente à prática^(3-4)^; conjuntura essa em que a simulação virtual passa a ser caracterizada como indispensável para alcançar tal necessidade.

A simulação virtual é tida como estratégia educacional que suscita a reprodução aproximada de situações reais em um ambiente controlado, o que corrobora o desenvolvimento de competências indispensáveis para a proposição da prática clínica^(5-6)^. Em uma perspectiva histórica, relativamente aos primeiros métodos de ensino adotados na educação em enfermagem, sabe-se que os estudantes desenvolviam suas competências clínicas mediante a realização de procedimentos diretamente em seus pares ou em pacientes. Todavia, tem-se que essa prática ocasiona riscos entre os estudantes bem como aos pacientes inseridos nos cenários de cuidados implicados na educação de profissionais de saúde^(7)^. Na medida em que a enfermagem avança enquanto profissão e ciência, o desenvolvimento tecnológico intrínseco corrobora o progresso da educação e, com isso, práticas rudimentares de ensino deixam de ser adotadas em diferentes contextos^(7-8)^.

No que diz respeito à simulação virtual, dentre suas diferentes modalidades, destaca-se a simulação clínica como método de ensino e aprendizagem bem-sucedido para a composição da qualificação da educação em enfermagem, visto que oportuniza aos estudantes a prática de suas competências clínicas sem prejuízos e riscos aos pacientes^(9-10)^. A aplicação dessas estratégias favorece a autoconfiança e a satisfação de estudantes de enfermagem desde suas primícias educacionais no decurso da graduação e perpassa a evolução da educação dessa profissão^(8,11-12)^, o que revela a imprescindibilidade da inclusão de ambientes de simulação clínica no processo formativo^(13)^.

Destarte, ainda que evidências científicas demonstrem a efetividade da adoção da simulação clínica na educação em enfermagem para o alcance de resultados positivos de aspectos inerentes à prática clínica (especialmente por se constituir como estratégia educacional que proporciona a transição da teoria para a prática)^(14-15)^, deve-se considerar que, apesar da heterogeneidade na educação em enfermagem na América Latina e Caribe, é notório que muitas Instituições de Ensino Superior (IES) ainda não utilizam a simulação clínica no processo formativo^(16)^. Não obstante, tal realidade é ainda mais agravante ao ponderarmos a inserção da simulação virtual nesse contexto, visto que, embora contribua para a qualificação do ensino clínico, apresenta alto custo, tornando sua implementação e sustentabilidade desafios para a educação em enfermagem.

De acordo com a Comissão Econômica para a América Latina e o Caribe (CEPAL), o ano de 2023 foi prospectado como desafiador para os países circunscritos à região supracitada, tendo em vista o baixo crescimento econômico ocasionado em virtude da alta desigualdade e má administração, que reduzem a confiança da população^(17)^. Nessa conjuntura, faz-se crucial considerar que, em tais países, o baixo crescimento econômico afeta diretamente a redução dos investimentos em educação e, consequentemente, o acesso às tecnologias para que a simulação virtual possa ser, de fato, empreendida.

À vista disso, reitera-se a relevância da simulação virtual para a educação em enfermagem para o alcance de resultados positivos no que diz respeito ao desenvolvimento dos conhecimentos, habilidades e atitudes de estudantes de enfermagem. Portanto, tendo em vista a imprescindibilidade de se conhecer o panorama e as tendências da simulação virtual para o avanço da educação em enfermagem na era digital, o presente estudo tem como objetivo examinar as características da produção científica da América Latina e Caribe a respeito da simulação virtual na educação em enfermagem.

## Método

### Tipo de estudo

Estudo bibliométrico realizado em cinco etapas, nomeadamente: delineamento de pesquisa, compilação de dados bibliométricos, análise, visualização e interpretação^(18)^; e reportado a partir das recomendações do *Preferred Reporting Items for Bibliometric Analysis* (PRIBA)^(19)^.

A este estudo bibliométrico, atribuíram-se os métodos de acoplamento bibliográfico e análise copalavra^(18)^. Assim, considerando o cenário da América Latina e Caribe, foram estabelecidas as seguintes perguntas de pesquisa:

Como se deu a produção científica a respeito da simulação virtual na educação em enfermagem ao longo do tempo?

Quais são os periódicos científicos que publicaram a respeito da simulação virtual na educação em enfermagem?

Quais são as modalidades de simulação virtual na educação em enfermagem?

Como ocorreu a coocorrência das palavras-chave em estudos relacionados à simulação virtual na educação em enfermagem?

### Fonte de dados

O estudo foi realizado na base de dados Literatura Latino-Americana e do Caribe em Ciências da Saúde (LILACS) ao se constituir como a mais importante e abrangente base de dados especializada na área da saúde, com literatura científica e técnica de 26 países da América Latina e do Caribe com acesso livre e gratuito. Tal base de dados abarca cerca de 1 milhão de registros distribuídos em 910 periódicos, ao longo de uma história de 38 anos^(20)^. Em comparação com outras bases de dados, como a *Medical Literature Analysis and Retrieval System Online databases* via *TheNational Library of Medicine* (MEDLINE), *Cumulative Index to Nursing and Allied Health* (CINAHL), *Web of Science*, Embase e Scopus, embora não excludentes, tem**-**se que apenas a LILACS é sensível ao contexto em estudo, notadamente na investigação acurada do panorama da produção científica sobre simulação virtual na educação em enfermagem na América Latina e Caribe, dada sua especificidade em abarcar o maior acervo científico circunscrito à região.

### Critérios de elegibilidade

Foram incluídos estudos que atenderam aos seguintes critérios: 1) estudos primários sobre simulação virtual na educação de estudantes de graduação em enfermagem; 2) estudos oriundos de países da América Latina e Caribe; e 3) estudos publicados nos idiomas português, espanhol e inglês (língua de proficiência dos autores). Foram excluídos estudos conduzidos sob a ótica de educadores de enfermagem a respeito da simulação virtual. Destaca-se que, para este estudo bibliométrico, não foi adotado recorte temporal. Todavia, foi estipulada seleção de estudos publicados até o ano de 2022, considerando-o como último ano completo durante o desenvolvimento do estudo, o que viabiliza a análise bibliométrica com foco na dinâmica; além disso, a literatura cinzenta não foi considerada.

### Estratégia de busca

Em abril de 2023, foi conduzida uma busca-piloto a fim de identificar os termos cadastrados no Descritores em Ciências da Saúde (DeCS) mais adequados para a elaboração da estratégia de busca. A busca foi realizada em 05 de maio do mesmo ano, mediante a seguinte estratégia de busca: ((mh:(“Realidade Virtual”)) OR (mh:(“*RealidadVirtual*”)) OR (mh:(“*Virtual Reality*”)) OR (“Realidade Virtual”) OR (“*RealidadVirtual*”) OR (“*Virtual Reality”*) OR (mh:(”Treinamento por Simulação”)) OR (mh:(“*Computer Simulation*”)) OR (mh:(“*Entrenamiento Simulado*”)) OR (“Treinamento por Simulação”) OR (“*Computer Simulation*”) OR (“*Entrenamiento Simulado*”) OR (mh:(“Simulação por Computador”)) OR (mh:(“*Computer Simulation*”)) OR (mh:(“*Simulación por Computador*”)) OR (“Simulação por Computador”) OR (“*Computer Simulation*”) OR (“*Simulación por Computador*”) OR (“Simulação Virtual”) OR (“*Simulación Virtual*”) OR (“*Virtual Simulation*”)) AND ((mh:(“Educação em Enfermagem”)) OR (mh:(“*Educación en Enfermería*”)) OR (mh:(“*Education, Nursing*”)) OR (mh:(“*Nursing Education*”)) OR (“Educação em Enfermagem”) OR (“*Educación en Enfermería*”) OR (“*Education, Nursing*”) OR (“*Nursing Education*”) OR (mh:(“Educação”)) OR (mh:(“*Educación*”)) OR (mh:(“*Education*”)) OR (Educa*) OR (“*Educación*”) OR (“*Education*”) OR (mh:(“Enfermagem”)) OR (mh:(“*Enfermería*”)) OR (mh:(“*Nursing*”)) OR (“Enfermagem”) OR (“*Enfermería*”) OR (“*Nursing*”) OR (Enfermeir*) OR (Nurse*) OR (Enfermer*)). Ressalta-se que a estratégia de busca foi desenvolvida com o suporte de uma bibliotecária universitária com experiência.

Com o propósito de reduzir a inclusão de estudos divergentes ao método proposto (especialmente no que diz respeito aos *outliers*, isto é, estudos que se diferenciam significativamente dos demais, na medida em que sua presença pode reduzir a validade dos resultados), os estudos localizados foram exportados para a plataforma *Rayyan* para seleção, de forma independente e mascarada, por dois revisores^(21)^, em acordo ao referencial metodológico^(18)^. O processo de triagem, elegibilidade e inclusão dos estudos ocorreu a partir da leitura de títulos e resumos, tendo, como caráter norteador, o objetivo e as perguntas de pesquisa. A divergência na análise foi resolvida por um terceiro revisor com expertise no tema que auxiliou na seleção final dos estudos que compuseram a amostra.

### Indicadores bibliométricos

A amostra final foi exportada para o editor de planilhas *Microsoft Excel* ^®^, para análise descritiva dos estudos selecionados. Em seguida, procedeu-se à análise bibliométrica no *VOSviewer*, *version* 1.6.20. A escolha de tal *software* deu-se mediante as diversas possibilidades de análise, uma vez que permite a construção de redes bibliométricas que podem incluir periódicos, grupos de pesquisadores, redes de citação, redes de autoria, co autoria e grupos de palavras mais prevalentes^(22)^. Enfatiza-se que as palavras-chave e suas variações foram apresentadas em apenas um formato, ou seja, a palavra raiz foi substituída pela sua versão mais completa, considerando o seu cadastro no DeCS. Tal estratégia foi aplicada para evitar duplicidade de dados na amostra final, conforme recomendado pelo referencial metodológico^(18)^.

Para analisar os dados bibliométricos, duas leis da bibliometria foram utilizadas: a Lei de Bradford e a Lei de Zipf. A Lei de Bradford examina a produtividade dos periódicos, permitindo classificar sua relevância com base na quantidade de artigos publicados sobre um tema específico. Por outro lado, a Lei de Zipf mensura a frequência das palavras nos textos, possibilitando sua ordenação e análise de acordo com o foco da pesquisa^(23)^. Além disso, foram consideradas a evolução anual das publicações e a modalidade de simulação empregada nos artigos publicados na América Latina e no Caribe sobre simulação virtual na educação em enfermagem.

A análise descritiva da produção científica sobre a simulação virtual na educação em enfermagem ao longo do tempo foi apresentada. Assim, detalhou-se a respeito da quantidade de publicações, segundo: ano de publicação, periódicos científicos e modalidades de simulação. Ademais, para a análise bibliométrica da coocorrência das palavras-chave, os dados foram apresentados em mapas semânticos, a fim de detalhar a estrutura cognitiva do campo proposto para o escopo deste estudo.

Reitera-se o propósito do estudo bibliométrico para a descrição de um campo^(18)^, definido, nesta pesquisa, como a simulação virtual na educação em enfermagem na América Latina e Caribe. Assim, realizou-se análise com foco na dinâmica, de modo a interpretar os anos de publicação dos estudos, a partir de uma linha temporal que ponderou eventos que justificam o aumento ou redução da produção sobre simulação virtual na educação em enfermagem. Ainda, analisaram-se os periódicos que apresentaram maior quantitativo de publicações sobre a temática, o que pode ser relevante para pesquisadores com interesse em simulação virtual na educação em enfermagem.

Utilizou-se a abordagem analítica para a interpretação da estrutura dos estudos selecionados. Por meio disso, foi possível compreender as relações entre os elementos estruturais (periódicos científicos e modalidades de simulação) e suas repercussões no campo do escopo proposto. Na sequência, o mapa semântico foi definido segundo os *clusters* da colaboração de coocorrência das palavras-chave. Para a análise de coocorrência das palavras-chave, o *VOSviewer* foi configurado para considerar, no mínimo, duas ocorrências para uma mesma palavra-chave.

## Resultados

Ao total, 579 estudos foram localizados, dos quais 57 foram selecionados para leitura na íntegra. Após a aplicação dos critérios de elegibilidade, 46 estudos compuseram a amostra final^(24-69)^, conforme evidenciado na Figura 1.

**Figura 1 -:** *Preferred Reporting Items for Bibliometric Analysis* (PRIBA)

Assim, este estudo bibliométrico analisou 46 artigos científicos sobre simulação virtual na educação em enfermagem na América Latina e Caribe. A amostra apresenta a média de seis autores por publicação, sendo notório o crescimento da produção científica nesta área, que passou de uma média de 0,17 artigos por ano entre 1994 e 2016 e 4,33 entre 2017 e 2019 para 9,6 de 2020 até 2022, conforme ilustra a Figura 2.

**Figura 2 -:** Número de artigos publicados na América Latina e Caribe sobre simulação virtual na educação em enfermagem por ano. Ribeirão Preto, SP, Brasil, 2023

Entre os países da América Latina e Caribe, apenas quatro realizaram pesquisas a respeito do tema. O Brasil foi o país que mais desenvolveu estudos, correspondendo a ~84,78% (n=39)^(24-39,41-51,53-62,64-65)^ do total da amostra, seguido pelo Chile, com ~6,52% (n=3)^(63,66-67)^, Colômbia, com ~4,35% (n=2)^(40,52)^, e Cuba, com 4,35% (n=2)^(68-69)^.

A Figura 3 apresenta os periódicos que mais publicaram estudos relacionados ao tema, predominantemente de origem brasileira, destacando-se a Revista Latino-Americana de Enfermagem com 17,39% (n=8)^(27,36,38,45,49,60,62,64)^, a Revista Brasileira de Enfermagem com 15,29% (n=7)^(24,30-31,35,47,54,57)^ e a Escola Anna Nery com 13,04% (n=6)^(25,29,33,41,43,53)^.

A partir de um *framework* conceitual^(70)^, os cenários identificados na amostra foram classificados segundo as modalidades de simulação (Figura 4).

**Figura 3 -:** Periódicos de publicação dos artigos originados na América Latina e Caribe sobre simulação virtual na educação em enfermagem. Ribeirão Preto, SP, Brasil, 2023

**Figura 4 -:** Modalidade da simulação empregada nos artigos publicados na América Latina e Caribe sobre simulação virtual na educação em enfermagem. Ribeirão Preto, SP, Brasil, 2023

A estrutura cognitiva foi definida pelos autores por meio de palavras-chave que sintetizavam o enfoque de seus estudos. Assim, verificaram-se 272 palavras-chave, agrupadas de acordo com a coocorrência, com no mínimo duas menções. Conforme ilustra a Figura 5, a rede bibliométrica representa a coocorrência das palavras-chave, mostrando o número de termos em comum por meio de círculos e linhas. O tamanho dos círculos e a espessura das linhas são proporcionais ao número de termos citados, de modo que as conexões entre os termos indicam suas relações.

**Figura 5 -:** Análise de coocorrência das palavras-chave pelos autores dos estudos publicados na América Latina e Caribe sobre simulação virtual na educação em enfermagem. Ribeirão Preto, SP, Brasil, 2023

Dessa forma, observou-se que o termo “treinamento por simulação” (n=38) apresentou maior frequência e força de *link*, conectado a outros termos como, como “educação em enfermagem” (n=27), “estudantes de enfermagem” (n=14) e “enfermagem” (n=14). Outras áreas destacadas incluem: “competência clínica” (n=4), “simulação de paciente” (n=4), “educação” (n=4) e “segurança do paciente” (n=4). A imagem evidencia a relevância do treinamento prático e simulado no desenvolvimento de habilidades essenciais para a prática clínica em enfermagem, bem como sua interseção com métodos de ensino inovadores e a segurança do paciente.

## Discussão

O aumento progressivo de publicações relacionadas ao tema, em face de um contexto expressivo, América Latina e Caribe, pode ser consequência de fenômenos globais, seja em caráter processual seja emergencial. O primeiro diz respeito ao amadurecimento coletivo de uma profissão ou área de conhecimento em direção aos objetos de interesse que lhes são caros. Já o segundo está relacionado às questões emergentes e circunstanciais que podem afetar a aparente ordem natural dos processos sociais, o que inclui o padrão de publicação científica.

No âmbito operacional, tem-se que resultados científicos revelam a necessidade de que haja maiores e melhores conexões entre a realidade prática e a abordagem teórica no processo ensino-aprendizagem. Desse modo, espera-se que as produções científicas avancem para o desenvolvimento de melhores evidências sobre mecanismos e estratégias facilitadoras de uma formação coerente com as demandas sociais já percebidas e problematizadas no decurso da formação do estudante, o que não exclui a realidade da saúde e, por conseguinte, da enfermagem^(71-72)^.

Na formação do enfermeiro, quando associada a metodologias ativas, a simulação virtual se configura como valiosa estratégia para conectar o mundo real e virtual durante o processo ensino-aprendizagem do estudante, o que corrobora a performance do estudante e o alcance de objetivos por parte dos docentes e das instituições formadoras. A simulação virtual possibilita a transposição dos estudantes para realidades totalmente paralelas e virtuais geradas por inteligência computacional, em que podem, de maneira segura, explorar e viver experiências fora da realidade objetiva. Tais experiências estimulam sentidos, tais como, visão, audição e, por vezes, até mesmo o tato e olfato. Depreende-se dessa realidade, por suposto, o movimento de interesse da enfermagem e demais áreas de conhecimento pelas pesquisas sobre simulação clínica^(4)^.

As simulações para o ensino podem variar em termos de contexto (suporte básico de vida, atendimento de enfermagem à mulher e visita domiciliar) e nível de imersão. Neste estudo, foram analisadas as simulações virtuais, tais como, a telessimulação, os *serious games*, a realidade virtual imersiva, dentre outras. Outra modalidade foi a imersão clínica simulada, modelo adotado com maior frequência que recria cenários clínicos com alto grau de realismo, envolvendo o cuidado de pacientes, equipamentos complexos, atores e expressivo fluxo de dados^(70)^.

Outra forma de simulação é a processual, que permite a prática de habilidades técnicas específicas em procedimentos determinados, como o cateterismo venoso periférico e a administração segura de medicamentos e vacinas. Por fim, há o paciente simulado, em que um ator interpreta o papel de um paciente real para o treinamento e manejo de necessidades e condições clínicas e emocionais. Tais estratégias são eficazes para o desenvolvimento de aspectos atitudinais na qualidade de aptidões cognitivas, comportamentais e psicomotoras, e aspectos afetivos dos estudantes em ambiente simulado^(73)^. Nesse sentido, com a necessidade do desenvolvimento de uma cultura de inovação em toda a infraestrutura acadêmica, o ambiente de ensino tem o potencial de inspirar o cultivo de liderança, competência primordial para a enfermagem^(74)^.

Em se tratando das questões emergentes e circunstanciais que afetam as dinâmicas sociais, os dados desta pesquisa foram sensíveis ao expressivo aumento de estudos publicados durante a pandemia COVID-19. Tal conjuntura evidenciou a necessidade de melhores adaptações tecnológicas para a manutenção do processo formativo. Como consequência, tem-se a necessidade de melhores respostas pautadas na ciência e celeridade nas produções científicas. Assim, reitera-se a dinâmica da publicação científica como processo em constante evolução moldado pela interação entre avanços tecnológicos, demandas da sociedade e as práticas estabelecidas na comunidade^(75)^.

Líder nas produções sobre simulação virtual no contexto da América Latina e Caribe, o Brasil obteve um crescimento de 32,2% de artigos científicos no ano de 2020 em relação ao ano de 2015, como constatam os dados consolidados pelo Observatório de Ciência, Tecnologia e Inovação (OCTI) bem como pelo Centro de Gestão e Estudos Estratégicos (CGEE)^(76)^. Em acordo aos resultados, este estudo evidenciou que as produções brasileiras são majoritárias, atingindo mais de 80%. Essa realidade pode ser resultante das demandas de um país continental bem como de sua estrutura formativa no âmbito da pós-graduação, posto que possui 39 cursos de doutorado em enfermagem, ao passo que o Chile (país que aparece em segundo lugar em termos de produção sobre simulação virtual) apresenta dois cursos de doutorado. Esse panorama é atestado pelo entendimento de que a produção científica é, sobretudo, desenvolvida no contexto das universidades e em suas pós-graduações *stricto sensu* ^(77)^.

A aparente pluralidade de periódicos científicos que apresentaram produções sobre simulação virtual (identificados, em nosso estudo, em um total de 21 revistas), não condiz, contudo, com a realidade da América Latina e Caribe, visto que não retrata nem se aproxima da totalidade dos periódicos desta região. A título de ilustração numérica, a Rede Ibero-Americana de Editoração Científica em Enfermagem (RedEDIT) congrega 72 periódicos que são editados na América Latina. Destaca-se que tal número de periódicos não representa a totalidade de revistas dessa região, uma vez que nem todas estão associadas à rede supracitada^(78)^. Ademais, a concentração das publicações em somente três periódicos demonstra a imprescindibilidade de investimentos em pesquisa científica como incentivo ao desenvolvimento de evidências relacionadas à simulação virtual em enfermagem.

A rede bibliométrica conforma importante mecanismo imagético da realidade investigada. Sendo assim, os termos “treinamento por simulação”, “educação em enfermagem” e “estudantes de enfermagem”, embora prospectados na temática intencionada neste estudo, revelam a natureza problematizada do objeto, pois demonstram a importância de estratégias que associam tecnologias e princípios da metodologia ativa em direção ao processo de ensino-aprendizagem capazes de provocar novas experiências ao estudante, pautadas na realidade do cuidado ao paciente que é permeada por incertezas e riscos. Em campos periféricos da rede bibliométrica, é possível perceber termos relacionados a conhecimentos específicos relativos às práticas avançadas, por exemplo. Nessa conjuntura, estão termos “reanimação cardiopulmonar”, “competência clínica”, “enfermagem pediátrica” e “segurança do paciente”. Assim, o mapa semântico evidencia a importância do treinamento por simulação, além de abordar temas correlatos, apontando outros conceitos emergentes que têm potencial para moldar a educação em enfermagem no futuro.

Não obstante, ainda que a principal base de dados da América Latina e Caribe tenha sido utilizada neste estudo, entende-se que alguns periódicos científicos da região não possuem indexação na referida base de dados, o que limitou a identificação de potenciais estudos. No mais, destaca-se a não inclusão do idioma francês como uma limitação. Ainda que tal idioma apresente relevância para a região estudada, os autores não poderiam acessá-los, já que não possuem proficiência. Possíveis estudos em francês poderiam fornecer informações a respeito da produção científica da América Latina e Caribe a respeito da simulação virtual na educação em enfermagem.

Embora o estudo esteja relacionado à educação em enfermagem no âmbito da graduação, portanto, em direção ao processo de formação generalista, a simulação virtual pode conferir melhores possibilidades de aprofundamentos teórico e técnico-científico para conhecimentos específicos da prática profissional do enfermeiro. Assim, apesar das disparidades na produção científica, tal prática deve ser recomendada no contexto da educação em enfermagem em prol do avanço da profissão.

## Conclusão

O panorama da produção científica a respeito da simulação virtual na educação em enfermagem em países da América Latina e Caribe foi evidenciado, destacando-se predominantemente a produção científica de origem brasileira, em cujo contexto a Revista Latino-Americana de Enfermagem (RLAE) assume a liderança. Os resultados obtidos reforçam o entendimento do “treinamento por simulação” como valiosa estratégia para conectar teoria e prática durante o processo ensino-aprendizagem, corroborando o desenvolvimento de competências em estudantes de enfermagem. O estudo ressalta a importância da pesquisa em simulação virtual para a educação em enfermagem, destacando tendência crescente no desenvolvimento de estudos relacionados à simulação virtual com foco na imersão clínica simulada. Ademais, enfatiza as oportunidades significativas para colaboração internacional e compartilhamento de recursos entre pesquisadores, promovendo uma abordagem abrangente e colaborativa para avanços nesse campo. Contudo, tem-se os aspectos econômicos como principal limitação para implementação e sustentabilidade da simulação virtual na educação em enfermagem bem como para o desenvolvimento de novas evidências científicas que possam diagnosticar os diferentes cenários e contextos.

# Translation: Simulación virtual en la educación en enfermería en América Latina y el Caribe: estudio bibliométrico*

**DOI:** 10.1590/1518-8345.7261.4421

**Authors:** Agostinho A.C. Araújo, Lucas Gardim, Sara Soares dos Santos, Ítalo Rodolfo Silva, Manoel Carlos Neri da Silva, Simone de Godoy, Isabel Amélia Costa Mendes

**Affiliations:** 1 Universidade de São Paulo, Escola de Enfermagem de Ribeirão Preto, Centro Colaborador de la OPS/OMS para el Desarrollo de la Investigación en Enfermería, Ribeirão Preto, SP, Brasil; 2 Becario del Conselho Nacional de Desenvolvimento Científico e Tecnológico (CNPq), Brasil; 3 University of Alberta, Faculty of Nursing, Edmonton, AB, Canadá; 4 Universidade Federal do Rio de Janeiro, Escola de Enfermagem Anna Nery, Rio de Janeiro, RJ, Brasil; 5 Conselho Federal de Enfermagem, COFEn, Brasília, DF, Brasil

**Keywords:** Educación en Enfermería, Enfermería, Enseñanza, Estudiantes de Enfermería, Realidad Virtual, Entrenamiento Simulado

## Abstract

examinar las características de la producción científica en América Latina y el Caribe sobre simulación virtual en educación en enfermería.

estudio bibliométrico realizado en cinco etapas: diseño de la Investigación, compilación de datos bibliométricos, análisis, visualización e interpretación; en informe basado en las recomendaciones de *Preferred Reporting Items for Bibliometric Analysis* (PRIBA). Búsqueda realizada en Literatura Latinoamericana y del Caribe en Ciencias de la Salud (LILACS). Para el análisis bibliométrico se utilizó VOSviewer versión 1.6.20. Para interpretar datos se aplicaron las Leyes de Bradford y Zipf, considerando la dinámica temporal de publicaciones, abordaje analítico de la estructura de los estudios seleccionados y sus repercusiones en la simulación virtual de la enseñanza de enfermería en América Latina y el Caribe.

fueron hallados 579 estudios, 46 de ellos integraron la muestra final. Los estudios incluidos fueron publicados entre 1994 y 2022, y solo Brasil, Chile, Colombia y Cuba realizaron investigaciones relacionadas con la simulación virtual. El mapa semántico dio como resultado cinco *clusters*, destacándose “entrenamiento simulado” como el término más fuerte.

existe una tendencia creciente en el desarrollo de estudios relacionados con la simulación virtual en la educación en enfermería en América Latina y el Caribe.

## Destacados

**(1)** La simulación virtual conecta la teoría y la práctica en la educación en enfermería.**(2)** Solo Brasil, Chile, Colombia y Cuba realizaron investigaciones sobre el tema. **(3)** Las revistas científicas brasileñas lideran la producción científica sobre simulación virtual. **(4)** Hubo un incremento en la producción científica relacionada con la temática.

## Introducción

Es fundamental que los estudiantes, desde su educación de grado, desarrollen las competencias necesarias para practicar la atención de salud con calidad y seguridad^(1-2)^. En lo que respecta a la enfermería, considerando el creciente progreso de las tecnologías de la salud, se espera una mayor inversión en la pedagogía subyacente a la práctica^(3-4)^; circunstancia en la que la simulación virtual se revela como indispensable para alcanzar dicha necesidad.

La simulación virtual es entendida como un método educativo que permite la reproducción aproximada de situaciones reales en un ambiente controlado, corroborando el desarrollo de competencias imprescindibles para la propuesta de la práctica clínica^(5-6)^. Desde una perspectiva histórica, en relación con los primeros métodos de enseñanza adoptados en la educación en enfermería, se sabe que los estudiantes desarrollaban sus competencias clínicas realizando procedimientos directamente sobre sus pares o en pacientes. Sin embargo, es sabido que esta práctica provoca riesgos entre los propios estudiantes, así como a los pacientes presentes en los escenarios de atención implicados en la educación de los profesionales de salud^(7)^. A medida que la enfermería avanza como profesión y como ciencia, el desarrollo tecnológico intrínseco demuestra el progreso de la educación y, como consecuencia, dejan de adoptarse prácticas rudimentarias de enseñanza en diferentes contextos^(7-8)^.

Con respecto a la simulación virtual, entre sus diferentes modalidades, la simulación clínica se destaca como un método de enseñanza y aprendizaje exitoso para la calificación de la educación de enfermería, dado que brinda a los estudiantes la oportunidad de practicar sus competencias clínicas sin dañar o poner en riesgo a los pacientes^(9-10)^. La aplicación de estas estrategias favorece la autoconfianza y satisfacción de los estudiantes de enfermería desde sus inicios educativos, durante sus estudios de grado, e impregna la evolución de la educación en esta profesión^(8,11-12)^, lo cual revela la indispensabilidad de incluir entornos de simulación clínica en el proceso de formación^(13)^.

Así, aunque las evidencias científicas demuestren la efectividad de adoptar la simulación clínica en la educación en enfermería para alcanzar resultados positivos en aspectos inherentes a la práctica clínica (especialmente por constituir una estrategia educativa que permite la transición de la teoría a la práctica)^(14-15)^, debe considerarse que, a pesar de la heterogeneidad de la educación en enfermería en América Latina y el Caribe, es notorio que muchas Instituciones de Educación Superior (IES) aún no utilizan la simulación clínica en sus procesos de formación^(16)^. Sin embargo, esta realidad es aún más grave si consideramos la inclusión de la simulación virtual en este contexto, ya que aunque contribuye a la calificación de la enseñanza clínica, su costo es elevado, lo que hace que su implementación y sostenibilidad constituyan un desafío para la educación en enfermería.

De acuerdo con la Comisión Económica para América Latina y el Caribe (CEPAL), el año 2023 fue pronosticado como desafiante para los países de la mencionada región, considerando el reducido crecimiento económico causado por la alta desigualdad y la mala administración, que reducen la confianza de la población^(17)^. En dicho contexto, resulta esencial tener en cuenta que, en estos países, el escaso crecimiento económico incide directamente en la reducción de la inversión en educación y, consecuentemente, en el acceso a las tecnologías para que la simulación virtual pueda emprenderse efectivamente.

En vista de ello, se reitera la importancia de la simulación virtual para la educación de enfermería y su influencia creíble en el logro de resultados positivos en cuanto al desarrollo de conocimientos, habilidades y actitudes de los estudiantes de enfermería. Por ello, dada la importancia de conocer el panorama y las tendencias de la simulación virtual en la educación de enfermería en el contexto de América Latina y el Caribe, el presente estudio tiene como objetivo examinar las características de la producción científica en América Latina y el Caribe respecto de la simulación virtual en la educación en enfermería.

## Método

### Tipo de estudio

Estudio bibliométrico realizado en cinco etapas, a saber: diseño de la investigación, compilación de datos bibliométricos, análisis, visualización e interpretación^(18)^; e informe basado en las recomendaciones de los *Preferred Reporting Items for Bibliometric Analysis* (PRIBA)^(19)^.

Para este estudio bibliométrico se aplicaron los métodos de acoplamiento bibliográfico y análisis de co-palabras^(18)^. Así, considerando el escenario de América Latina y el Caribe, se establecieron las siguientes preguntas de investigación:

¿Cómo evolucionó a lo largo del tiempo la producción científica sobre simulación virtual en la educación en enfermería?

¿Cuáles son las publicaciones científicas que incluyeron artículos sobre simulación virtual en la educación en enfermería?

¿Cuáles son las modalidades de simulación virtual en la educación en enfermería?

¿Cómo se dio la coocurrencia de las palabras clave en los estudios relacionados con la simulación virtual en la educación en enfermería?

### Fuente de dados

El estudio se realizó en la base de datos Literatura Latinoamericana y del Caribe en Ciencias de la Salud (LILACS), la cual es la “base de datos especializada más importante y completa en el área de salud, con literatura científica y técnica de 26 países de América Latina y el Caribe y de acceso libre y gratuito”. Esta base de datos incluye cerca de 1 millón de registros distribuidos en 910 revistas a lo largo de 38 años de historia^(20)^. En comparación con otras bases de datos, como la *Medical Literature Analysis and Retrieval System Online* de la *National Library of Medicine* (MEDLINE), *Cumulative Index to Nursing and Allied Health* (CINAHL), *Web of Science*, *Embase* y *Scopus*, aunque no resulten excluyentes entre sí, solo LILACS es sensible al contexto del estudio, especialmente en la investigación precisa del panorama de la producción científica sobre simulación virtual en la educación en enfermería en América Latina y el Caribe, dada su especificidad en abarcar el mayor acervo científico circunscripto a la región.

### Criterios de elegibilidad

Fueron incluidos los estudios que cumplieron los siguientes criterios: 1) estudios primarios sobre simulación virtual en la educación de estudiantes de pregrado en enfermería; 2) estudios de países de América Latina y el Caribe; y 3) estudios publicados en portugués, español e inglés (idiomas dominados por los autores). Quedaron excluidos los estudios en la perspectiva de educadores de enfermería sobre simulación virtual. Cabe destacar que este estudio bibliométrico no adoptó un recorte temporal. No obstante, se estableció seleccionar estudios publicados hasta 2022, considerándolo como el último año completo a lo largo del desarrollo del estudio, lo que permitió un análisis bibliométrico con foco en la dinámica. Además, no fue considerada la literatura gris.

### Estrategia de búsqueda

En abril de 2023 se practicó una búsqueda piloto, a efectos de identificar los términos registrados en Descriptores en Ciencias de la Salud (DeCS) más adecuados para la elaboración de la estrategia de búsqueda. La misma se realizó el 5 de mayo del mismo año, a través de la estrategia a continuación: ((mh:(“Realidade Virtual”)) OR (mh:(“Realidad Virtual”)) OR (mh:(“Virtual Reality”)) OR (“Realidade Virtual”) OR (“Realidad Virtual”) OR (“Virtual Reality”) OR (mh:(“Treinamento por Simulação”)) OR (mh:(“Computer Simulation”)) OR (mh:(“Entrenamiento Simulado”)) OR (“Treinamento por Simulação”) OR (“Computer Simulation”) OR (“Entrenamiento Simulado”) OR (mh:(“Simulação por Computador”)) OR (mh:(“Computer Simulation”)) OR (mh:(“Simulación por Computador”)) OR (“Simulação por Computador”) OR (“Computer Simulation”) OR (“Simulación por Computador”) OR (“Simulação Virtual”) OR (“Simulación Virtual”) OR (“Virtual Simulation”)) AND ((mh:(“Educação em Enfermagem”)) OR (mh:(“Educación en Enfermería”)) OR (mh:(“Education, Nursing”)) OR (mh:(“Nursing Education”)) OR (“Educação em Enfermagem”) OR (“Educación en Enfermería”) OR (“Education, Nursing”) OR (“Nursing Education”) OR (mh:(“Educação”)) OR (mh:(“Educación”)) OR (mh:(“Education”)) OR (Educa*) OR (“Educación”) OR (“Education”) OR (mh:(“Enfermagem”)) OR (mh:(“Enfermería”)) OR (mh:(“Nursing”)) OR (“Enfermagem”) OR (“Enfermería”) OR (“Nursing”) OR (Enfermeir*) OR (Nurse*) OR (Enfermer*)). Cabe destacar que la estrategia de búsqueda fue desarrollada con la ayuda de una bibliotecaria experimentada.

Con el objeto de reducir la inclusión de estudios que se apartaran del método propuesto (especialmente en lo que respecta a los valores atípicos, es decir, estudios que difieren significativamente de los demás en el sentido de que su presencia puede reducir la validez de los resultados), los estudios localizados fueron exportados a la plataforma Rayyan para su selección de forma independiente y a ciegas por parte de dos revisores^(21)^, estrategia también descrita en el referencial metodológico^(18)^. El proceso de filtrado, elegibilidad e inclusión de los estudios se basó en la lectura de los títulos y resúmenes, con el objetivo y las preguntas de investigación como principios orientadores. Las divergencias en el análisis fueron resueltas por un tercer revisor especializado en el tema, que colaboró en la selección final de los estudios que constituyeron la muestra.

### Indicadores bibliométricos

La muestra final fue exportada al gestor de planillas de cálculo *Microsoft Excel*®^ para un análisis descriptivo de los estudios seleccionados. A continuación, se realizó el análisis bibliométrico utilizando *VOSviewer* versión 1.6.20. Se eligió este *software* por sus diversas posibilidades de análisis, ya que permite la construcción de redes bibliométricas que pueden incluir revistas científicas, grupos de investigadores, redes de citación, redes de autoría, coautoría y grupos de palabras más prevalentes^(22)^. Cabe destacar que las palabras clave y sus variaciones fueron presentadas en un único formato, es decir, la palabra raíz fue sustituida por su versión más completa, considerando su registro en el DeCS. Esta estrategia se aplicó para evitar la duplicidad de datos en la muestra final, como lo recomienda el referencial metodológico^(18)^.

Para analizar los datos bibliométricos se utilizaron dos leyes de la bibliometría: la Ley de Bradford y la Ley de Zipf. La Ley de Bradford examina la productividad de las revistas científicas, lo que permite clasificar su relevancia en base al número de artículos publicados sobre un tema específico. Por su parte, la Ley de Zipf mide la frecuencia de las palabras en los textos, permitiendo ordenarlos y analizarlos en función del foco de la investigación^(23)^. Además, fueron consideradas la evolución anual de las publicaciones y la modalidad de simulación utilizada en los artículos publicados en América Latina y el Caribe sobre simulación virtual en la educación en enfermería.

Se presentó un análisis descriptivo de la producción científica sobre simulación virtual en la educación en enfermería a lo largo del tiempo. Se analizó la cantidad de publicaciones según el año de publicación, las revistas científicas y las modalidades de simulación. Además, para el análisis bibliométrico de la coocurrencia de palabras clave, los datos fueron presentados en mapas semánticos con el fin de detallar la estructura cognitiva del campo propuesto para el ámbito de este estudio.

Se reitera el propósito del estudio bibliométrico para describir un campo^(18)^, definido en esta investigación como simulación virtual en la educación de enfermería en América Latina y el Caribe. Fue así que se realizó un análisis enfocado en la dinámica, a fin de interpretar los años de publicación de los estudios a partir de una línea temporal que consideró los acontecimientos que justifican el aumento o la disminución de la producción de la simulación virtual en la educación en enfermería. También se analizaron las revistas científicas con mayor cantidad de publicaciones sobre el tema, lo cual puede ser relevante para los investigadores interesados en la temática de la simulación virtual en la educación en enfermería.

Se utilizó un abordaje analítico para interpretar la estructura de los estudios seleccionados. Ello permitió comprender las relaciones entre los elementos estructurales (revistas científicas y modalidades de simulación) y sus repercusiones en el ámbito del alcance propuesto. A continuación, se definió el mapa semántico en función de los *clusters* de la colaboración de coocurrencia de las palabras clave. Para analizar la coocurrencia de las palabras clave, se configuró *VOSviewer* para considerar al menos dos apariciones de la misma palabra clave.

## Resultados

En total se hallaron 579 estudios, de los cuales 57 fueron seleccionados para su lectura completa. Tras aplicarse los criterios de elegibilidad, 46 estudios integraron la muestra final^(24-69)^, como se evidencia en la Figura 1.

**Figura 1 -:** *Preferred Reporting Items for Bibliometric Analysis* (PRIBA)

El presente estudio bibliométrico analizó 46 artículos científicos sobre simulación virtual en educación en enfermería en América Latina y el Caribe. La muestra expresa un promedio de seis autores por publicación, y se observa un crecimiento notable de la producción científica en el área, pasando de un promedio de 0,17 artículos anuales entre 1994 y 2016 a 4,33 entre 2017 y 2019 y a 9,6 entre 2020 y 2022, como se observa en la Figura 2.

**Figura 2 -:** Cantidad de artículos publicados en América Latina y el Caribe sobre simulación virtual en educación enfermería, por año. Ribeirão Preto, SP, Brasil, 2023

De los países de América Latina y el Caribe, solo cuatro han realizado investigaciones sobre el tema. Brasil fue el país que más estudios realizó, concentrando un ~84,78% (n=39)^(24-39,41-51,53-62,64-65)^ del total de la muestra, seguido de Chile, con un ~6,52% (n=3)^(63,66-67)^, Colombia, con un ~4,35% (n=2)^(40,52)^, y Cuba, con un 4,35% (n=2)^(68-69)^.

La Figura 3 presenta las revistas científicas que publicaron más estudios sobre el tema, predominantemente de origen brasileño, destacándose la *Revista Latino-Americana de Enfermagem* (RLAE) con 17,39% (n=8)^(27,36,38,45,49,60,62,64)^, la *Revista Brasileira de Enfermagem* (REBEn) con 15,29% (n=7)^(24,30-31,35,47,54,57)^ y la *Escola Anna Nery* con 13,04% (n=6)^(25,29,33,41,43,53)^.

Partiendo de un marco conceptual^(70)^, los escenarios identificados en la muestra fueron clasificados según las modalidades de simulación (Figura 4).

**Figura 3 -:** Revistas científicas de publicación de los artículos generados en América Latina y el Caribe sobre simulación virtual en la educación en enfermería. Ribeirão Preto, SP, Brasil, 2023

**Figura 4 -:** Modalidad de simulación aplicada en los artículos publicados en América Latina y el Caribe sobre simulación virtual en la educación en enfermería. Ribeirão Preto, SP, Brasil, 2023

La estructura cognitiva fue definida por los autores a partir de palabras clave que resumen el enfoque de sus estudios. El resultado fueron 272 palabras clave, agrupadas según su coocurrencia, con por lo menos dos menciones. Como se ilustra en la Figura 5, la red bibliométrica representa la coocurrencia de palabras clave, mostrando la cantidad de términos en común a través de círculos y líneas. El tamaño de los círculos y el grosor de las líneas son proporcionales a la cantidad de repeticiones de los términos, por lo que las conexiones entre los mismo indican sus relaciones.

**Figura 5 -:** Análisis de coocurrencia de las palabras clave por parte de los autores de los estudios publicados en América Latina y el Caribe sobre simulación virtual en la educación en enfermería. Ribeirão Preto, SP, Brasil, 2023

Así, se observó que el término “entrenamiento simulado” (n=38) presentó mayor frecuencia y fuerza de vínculo, relacionado con otros términos como “educación en enfermería” (n=27), “estudiantes de enfermería” (n=14) y “enfermería” (n=14). Otras áreas destacadas incluyen: “competencia clínica” (n=4), “simulación de paciente” (n=4), “educación” (n=4) y “seguridad del paciente” (n=4). La imagen resalta la relevancia de la formación práctica y simulada en el desarrollo de habilidades esenciales para la práctica clínica de enfermería, así como su intersección con métodos de enseñanza innovadores y la seguridad del paciente.

## Discusión

El progresivo aumento de las publicaciones relacionadas al tema en el contexto de América Latina y el Caribe puede ser consecuencia de fenómenos globales, tanto de procedimiento como de emergencia. Los primeros se refieren a la maduración colectiva de una profesión o área de conocimiento orientada hacia objetos de interés que le son propios. Los segundos tienen que ver con cuestiones emergentes y circunstanciales que pueden afectar el aparente orden natural de los procesos sociales, lo cual incluye al estándar de publicación científica.

En el aspecto operativo se observa que los resultados científicos revelan la necesidad de mayores y mejores conexiones entre la realidad práctica y el abordaje teórico en el proceso de enseñanza-aprendizaje. De esta forma, se espera que las producciones científicas avancen hacia el desarrollo de mejores evidencias sobre mecanismos y estrategias que faciliten una capacitación que se corresponda con las demandas sociales ya percibidas y problematizadas en el transcurso de la formación del estudiante, lo que no excluye a la realidad de la salud y, consecuentemente, de la enfermería^(71-72)^.

En la formación de enfermería, cuando está asociada a metodologías activas, la simulación virtual constituye una valiosa estrategia para conectar los mundos real y virtual durante el proceso de enseñanza-aprendizaje de los estudiantes, lo que corrobora el rendimiento de los alumnos y la consecución de objetivos por parte de docentes e instituciones formativas. La simulación virtual permite transportar a los estudiantes a realidades totalmente paralelas y virtuales generadas por la inteligencia computacional, en las que pueden explorar y vivir de forma segura experiencias fuera de la realidad objetiva. Estas experiencias estimulan sentidos como la vista, el oído y a veces incluso el tacto y el olfato. Es a partir de esta realidad, por supuesto, que la enfermería y otras áreas del conocimiento se han interesado por la investigación de la simulación clínica^(4)^.

Las simulaciones para la enseñanza pueden variar en términos de contexto (soporte vital básico, atención de enfermería a mujeres y visitas domiciliarias) y nivel de inmersión. En este estudio fueron analizadas simulaciones virtuales, como telesimulación, *serious games*, realidad virtual inmersiva, etc. Otra modalidad fue la inmersión clínica simulada; un modelo adoptado con mayor frecuencia que recrea escenarios clínicos con un alto grado de realismo, involucrando la atención de pacientes, equipos complejos, actores y un significativo flujo de datos^(70)^.

Otra forma de simulación es la procedimental, que permite practicar habilidades técnicas específicas en procedimientos determinados, como el cateterismo venoso periférico y la administración segura de medicamentos y vacunas. Por último, existe el paciente simulado, en el que un actor interpreta el papel de un paciente real para entrenar y gestionar necesidades y condiciones clínicas y emocionales. Estas estrategias son eficaces para desarrollar aspectos actitudinales en la calidad de las aptitudes cognitivas, conductuales y psicomotoras, así como aspectos afectivos de los estudiantes en un ambiente simulado^(73)^. En este sentido, ante la necesidad de desarrollar una cultura de innovación en toda la infraestructura académica, el ámbito docente cuenta con potencial para inspirar la cultura del liderazgo; una competencia esencial para la enfermería^(74)^.

En lo que respecta a las cuestiones emergentes y coyunturales que afectan a las dinámicas sociales, los datos de esta investigación fueron sensibles al significativo aumento de estudios publicados durante la pandemia de COVID-19. Esta coyuntura evidenció la necesidad de mejores adaptaciones tecnológicas para el mantenimiento del proceso formativo. En consecuencia, es necesario mejorar las respuestas basadas en la ciencia y la celeridad de la producción científica. Así, se reitera la dinámica de la publicación científica como un proceso en constante evolución, moldeado por la interacción entre los avances tecnológicos, las demandas de la sociedad y las prácticas establecidas en la comunidad^(75)^.

Líder en la producción sobre simulación virtual en el ámbito de América Latina y el Caribe, Brasil registró un incremento del 32,2% de artículos científicos en 2020 en relación a 2015, según datos consolidados por el Observatorio de Ciencia, Tecnología e Innovación (OCTI) y el Centro de Gestión y Estudios Estratégicos (CGEE)^(76)^. Como se destacó en los resultados, este estudio mostró que las producciones brasileñas son mayoritarias, alcanzando más del 80%. Esto puede deberse a las exigencias de un país continental, así como a su estructura formativa a nivel de posgrado, dado que cuenta con 39 programas de doctorado en enfermería, en tanto que Chile (país que ocupa el segundo lugar en cuanto a producción sobre simulación virtual) cuenta solamente con dos programas de doctorado. Este panorama se comprueba al comprender que la producción científica se desarrolla principalmente en el ámbito de las universidades y sus programas de postgrado *stricto sensu* ^(77)^.

Sin embargo, la aparente pluralidad de revistas científicas que presentaron producciones sobre simulación virtual (identificados según nuestro estudio en un total de 21 publicaciones) no se corresponde con la realidad de América Latina y el Caribe, ya que no representa ni se acerca a la totalidad de las revistas de la región. A título de ilustración numérica, la Red Iberoamericana de Edición Científica en Enfermería (RedEDIT) agrupa a 72 publicaciones de América Latina. Cabe destacar que este número de revistas científicas no representa la totalidad de publicaciones de la región, pues no todas están asociadas a la mencionada red^(78)^. Asimismo, la concentración de publicaciones en apenas tres revistas demuestra la indispensabilidad de invertir en investigación científica como incentivo al desarrollo de evidencias relacionadas con la simulación virtual en enfermería.

La red bibliométrica constituye un importante mecanismo de imagen de la realidad investigada. Así, los términos “capacitación por simulación”, “educación en enfermería” y “estudiantes de enfermería”, aunque prospectados en la temática prevista en este estudio, revelan la naturaleza problematizada del objeto, pues demuestran la importancia de estrategias que asocian tecnologías y principios de la metodología activa con vistas a un proceso de enseñanza-aprendizaje capaz de generar nuevas experiencias para los estudiantes a partir de la realidad de la atención al paciente, permeada por incertidumbres y riesgos. En campos periféricos de la red bibliométrica, es posible observar términos relacionados a conocimientos específicos sobre prácticas avanzadas, por ejemplo. Entre ellos se encuentran los términos “reanimación cardiopulmonar”, “competencia clínica”, “enfermería pediátrica” y “seguridad del paciente”. Así pues, el mapa semántico evidencia la importancia de la capacitación con simulación, además de abordar temas relacionados, señalando otros conceptos emergentes que tienen potencial para moldear la educación en enfermería en el futuro.

Sin embargo, aunque en este estudio se haya utilizado la principal base de datos de América Latina y el Caribe, se entiende que algunas revistas científicas de la región no están indexadas en la misma, lo que limitó la identificación de potenciales estudios. A la vez, la no inclusión del idioma francés constituye otra limitación. Aunque esta lengua es relevante para la región estudiada, los autores no pudieron considerarla por no tener dominio de ella. Posibles estudios en francés podrían haber brindado mayor información sobre la producción científica en América Latina y el Caribe en relación a la simulación virtual en la educación en enfermería.

Aunque el estudio haya estado relacionado con la formación de grado en enfermería y, por lo tanto, con el proceso de formación generalista, la simulación virtual puede ofrecer mejores posibilidades de profundización teórica y técnico-científica de los conocimientos específicos de la práctica profesional del enfermero. Así, a pesar de las disparidades en la producción científica, dicha práctica debe ser recomendada en el contexto de la educación en enfermería en pro del avance de la profesión.

## Conclusión

Se analizó el panorama de la producción científica sobre simulación virtual en la enseñanza de enfermería en los países de América Latina y el Caribe, en el que predominó la producción científica de origen brasileño, donde la *Revista Latino-Americana de Enfermagem* (RLAE) asume el liderazgo. Los resultados obtenidos refuerzan la comprensión de la “entrenamiento simulado” como una estrategia valiosa para conectar la teoría con la práctica durante el proceso de enseñanza-aprendizaje, corroborando el desarrollo de competencias en los estudiantes de enfermería. El estudio destaca la importancia de la investigación en simulación virtual para la educación en enfermería, resaltando una tendencia creciente en el desarrollo de estudios relacionados con la simulación virtual enfocados en la inmersión clínica simulada. Asimismo, pone de relieve las significativas oportunidades de colaboración internacional y de intercambio de recursos entre investigadores, promoviendo un abordaje integral y colaborativo de los avances en este campo. Sin embargo, existen aspectos económicos que suponen la principal limitación para la implantación y sostenibilidad de la simulación virtual en la educación en enfermería, así como para el desarrollo de nuevas evidencias científicas que puedan diagnosticar diferentes escenarios y contextos.
